# The role of social capital in explaining mental health inequalities between immigrants and Swedish-born: a population-based cross-sectional study

**DOI:** 10.1186/s12889-016-3955-3

**Published:** 2017-01-25

**Authors:** Charisse M. Johnson, Mikael Rostila, Anna C. Svensson, Karin Engström

**Affiliations:** 1 Department of Public Health Sciences, Karolinska Institutet, Widerströmska Huset, Tomtebodavägen 18A, Stockholm, 171 65 Sweden; 2 Centre for Health Equity Studies, Stockholm University/Karolinska Institutet Sveavägen 160, Stockholm, 106 91 Sweden

**Keywords:** Mental health, Social capital, Immigrants, Refugees, Inequalities, Duration of residence, Reason for immigration, Sweden

## Abstract

**Background:**

Social capital may theoretically explain health inequalities between social groups, but empirical evidence is lacking. Some studies indicate that social capital may be particularly important for immigrant health. Nearly 16% of Sweden’s population are foreign-born immigrants and research has shown them to be susceptible to psychological distress, though significant variation has been found between groups. In this study, we investigate the following hypotheses: 1) if non-refugees have better mental health than Swedish-born, and refugees experience worse mental health than Swedish-born; 2) if mental health status converges with that of Swedish-born with longer duration of residence; and 3) if social capital mediates the effect of immigrant status on psychological distress for different immigrant groups as compared to Swedish-born.

**Methods:**

This cross-sectional study uses baseline data from the Stockholm Public Health Cohort and includes 50,498 randomly-selected individuals from Stockholm County in 2002, 2006, and 2010. Mental health was measured as psychological distress, using the 12-item General Health Questionnaire. Social capital was measured using indicators of bonding, bridging, and linking social capital. Both cognitive and structural aspects were measured for the latter two indicators. Mediation was tested using logistic regression and the Sobel test.

**Results:**

The results show that refugees generally had greater odds of psychological distress than non-refugees compared to their respective Swedish-born counterparts. Among immigrant men, both refugees and non-refugees had significantly greater odds of psychological distress than Swedish-born men. Only refugee women in Sweden 10 years or more had significantly greater odds of psychological distress compared to Swedish-born women. The mediation analysis demonstrated that indicators of social capital mediated the association for all immigrant men (except non-refugees in Sweden 3-9 years) and for refugee women in Sweden 10 years or more. While bonding social capital showed the greatest mediatory role among the three social capital types, adding them together had the strongest explanatory effect.

**Conclusions:**

Social capital explains differences in mental health for some immigrant groups, highlighting its role as a potentially important post-migration factor. Increased investment from policy-makers regarding how social capital can be promoted among new arrivals may be important for preventing psychological distress.

**Electronic supplementary material:**

The online version of this article (doi:10.1186/s12889-016-3955-3) contains supplementary material, which is available to authorized users.

## Background

There are over 200 million international migrants in the world today [1]. Nearly 16% of Sweden’s population is foreign-born immigrants [2] and research has shown some groups to be particularly susceptible to psychological distress [3]. As a heterogeneous population, some variance in mental health status between immigrant groups is attributed to differences in pre-migration factors, such as exposure to trauma due to war or pre-migration economic status [4–6]. Post-migration conditions are also critical factors affecting immigrant mental health. Porter & Haslam [6] showed that positive post-migration conditions could ameliorate the effect of pre-migration trauma on mental health. Furthermore, numerous studies have demonstrated that immigrants are disproportionately affected by mental ill-health on account of factors relating to the individual’s social position in the new society, such as socioeconomic status, gender, ethnicity, and culture [4, 7–9].

Psychosocial mechanisms have been identified as one of the pathways linking social position and health inequalities [10, 11]. One explanation for adverse health consequences of inequalities is that it crowds out social relations and social capital [12–14]. Social capital is defined e.g. as “features of social organization such as networks, norms, and social trust that facilitate coordination and cooperation for mutual benefit” [15] and has been suggested to explain health inequalities between social groups [12–14]. A systematic review by Uphoff et al., [14] found strong evidence that low social capital is linked to socioeconomic inequalities in health, but empirical support of social capital as an explanatory factor in this relationship is lacking. However, previous studies have indicated that social capital may be particularly important with regard to health inequalities between immigrant groups. For example, a Norwegian study by Dahl & Malmberg-Himonen [16] indicated that while social capital negligibly mediated the effect of socioeconomic position on self-rated health for their overall study population, it was important for immigrants. A Swedish study demonstrated that social capital explains inequalities in psychological health by both social class and immigrant status [17], though the latter was defined by an economic classification of country of birth and did not include individual indicators of migration status.

Social capital can be conceptualised as three different types — bonding, bridging, and linking social capital. The distinction between these three types of social capital is based on the degree of homogeneity between individuals connected through some social network in terms of their social identity and position in the social hierarchy. Bonding social capital refers to the strong ties between individuals that share a common social identity and frequently interact, such as family or close friends [18, 19], and is often intertwined with the concept of social support. Bridging social capital characterizes weaker social ties between groups of people that are socially heterogeneous and would not normally interact [18–20], often measured by membership in organizations or trust in neighbors. Individuals in bonding and bridging networks are on the same level of the social hierarchy, whereas linking social capital (e.g. voting and trust in social institutions) characterizes connections between individuals across authority gradients [20]. Each of these types of social capital has a structural and a cognitive dimension [21, 22]. At the individual level, structural social capital refers to behaviour and implies participation in social networks, associations and other forms of civic engagement [19]. Cognitive social capital refers to individual perceptions of trust and reciprocity, reinforced by norms, values and attitudes prevalent in society [22].

Each type of social capital has links to psychological mechanisms that influence mental well-being. Bonding social capital provides feelings of stability, predictability, belonging and security which can have positive effects on mental health [23, 24]. In a systematic review conducted by De Silva and colleagues [23], 10 of 18 studies found a negative association between cognitive and/or structural aspects of bonding social capital and common mental disorders while seven found no association and one found a positive association. The authors noted that the combined sample size of the studies that found a significant negative association was twice that of those that found no association. The study that found a positive association was set in an impoverished, inner-city neighborhood in the United States [25], echoing sentiments that bonding social capital is not always beneficial, as it can entail social pressures and dynamics that are detrimental to mental health [26]. The open networks that characterize bridging and linking social capital are generally at less risk for generating and disseminating negative health externalities such as these [17]. Accordingly, the same study found that bridging social capital was inversely associated with mental distress, supporting other findings that individuals accessing bonding but not bridging social capital are prone to common mental disorders [27]. Bridging and linking social capital are thought to promote mental health through enabling information exchange across social groups and access to external assets, widening the circle of trust and creating opportunities for social mobility [17]. Previous studies have generally demonstrated an inverse relationship between psychological distress and the cognitive aspect of bridging social capital [see for example 28–30]. Less consistency has been found regarding the structural aspect [28–30]. Fewer studies have looked at the relationship between mental health and linking social capital. However, a Swedish-based cohort study showed that low linking structural social capital predicted psychosis [31] and another found a significantly increased risk of psychological distress for individuals with low cognitive linking social capital [30].

Generally, those with higher social status have larger social networks, higher levels of trust and receive greater quantity and quality social recourses from their networks [16, 17, 32]. Immigrants are a particularly vulnerable group in this sense, as a considerable share of their social network is left in their home country. It may take several years to establish a comparable social network and confidence in a new society. Furthermore, immigrants in Sweden and their children often live in ethnically segregated and economically disadvantaged areas [33] that may have poorer social infrastructure, with fewer possibilities to meet and interact [34]. In light of these realities, bonding social capital has been identified as an important first step for new immigrants, as they try to rebuild strong social ties of familiar support in a new society [14, 35]. Subsequently, bridging and linking social capital and the development of more formal ties with the wider-society are seen by migrant groups as methods for integration, while politicians consider them mechanisms for achieving society-wide social cohesion [35]. Though the opportunity to pursue these ends would be expected to increase with time spent in the new society, it is likely easier for some groups depending upon their reason for immigration. For example, labour immigrants and students have formal environments that may help facilitate the development of social connections, whereas typically do not.

Like social circumstances, immigrant mental health can vary based on reason for immigration and duration of residence in the host country. Much mental health research has focused on non-refugee and refugee populations, but few studies [36] compare the two. A meta-analysis by Lindert and colleagues [37] looked at the combined results of 35 studies and determined that refugees had twice the prevalence of depression and anxiety as labour immigrants. Another meta-analysis of 59 studies demonstrated that refugees suffered moderately poorer mental health outcomes than non-refugees [4]. In Europe, some studies have shown support for the "healthy immigrant effect", in which newcomers initially manifest better health than the host population [38]. This phenomenon is considered more relevant to non-refugees, as those who choose to migrate are likely healthier than the general population. Furthermore, non-refugees who are not successful in a new country with regards to their migration expectations, would be more likely to return to their country of origin, further reflecting that those who stay are healthier. Even so, immigrant health is theorized to fluctuate in accordance with acculturative stress [39] and research suggests that it generally converges with the health of the host population over time. Regarding refugees, the association between duration of residence and mental health has also been investigated in relation to post-migration stress and the long-term effects of trauma. For example, a study on the long-term effects of trauma on Vietnamese immigrants to Australia found that the effect on mental health fades over time, though they persisted longer and stronger for those exposed to 3 or more traumatic events [40].

The aim of this study is to explore whether social capital can explain mental health inequalities between foreign-born immigrant groups and Swedish-born. Immigrants will be grouped according to their reason for immigration and duration of residence in Sweden. Based on previous research, we hypothesize that: 1) non-refugees have better mental health compared to Swedish-born while refugees are more likely to experience worse mental health; 2) immigrant mental health converges with that of Swedish-born with longer duration of residence; and 3) after adjustment for socioeconomic factors, social capital partially or fully explains differences in mental health between immigrants and Swedish-born. As gender differences in mental health have been well established, men and women are analysed separately.

## Methods

This is a cross-sectional study using data from the Stockholm Public Health Cohort (SPHC) initiated in 2002 [41]. Every 4 years, questionnaires were sent to a new cohort of 50,000-57,000 randomly selected Stockholm County residents (population in 2006: 1.9 million, 19% foreign-born immigrants, www.scb.se), stratified for sub-region, and follow-up questionnaires were sent to previous cohorts. The questionnaire included 100 questions on health status, social circumstances, and other lifestyle factors. Questionnaire data were supplemented with individually-linked registry information from the National Board of Health and Welfare and Statistics Sweden [described in 41] and is detailed in the following subsections. Data were weighted to adjust for stratified sampling and non-response related to e.g. sex, age, country of birth, income, educational level, and area of residence. Questionnaires were minimally modified from cohort to cohort. In addition to Swedish, questionnaires for the 2006 cohort were available in Arabic, English, Farsi, Finnish, Spanish, and Turkish [41, 42].

### Study sample

**Fig. 1:**
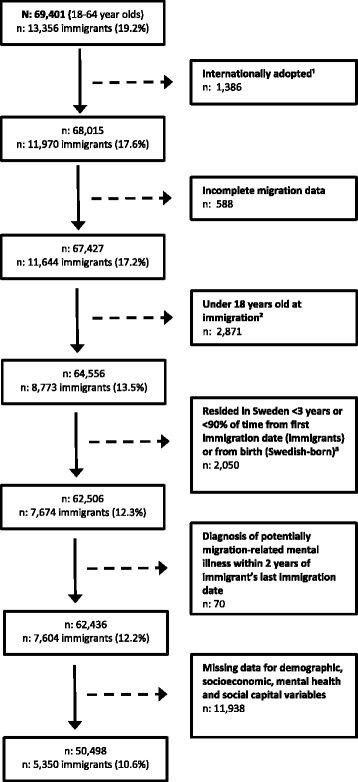
Participants excluded and lost in creating the final study sample. ^1^Internationally adopted individuals were considered ineligible for inclusion. ^2^Individuals are considered adults at age 18 in Sweden. ^3^This exclusion was made based on the specifications of the social capital measure. Linking social capital measures participation in elections and non-European immigrants can only vote in municipal and county elections after three years of residence in Sweden. Additionally, individuals who spent less than 90% of the time in Sweden were excluded on account of ensuring to the extent possible that the social capital being measured reflects experiences and feelings toward Sweden and not elsewhere

The 2002, 2006 and 2010 baseline SPHC data were pooled for this study. Response rates for individuals aged 18–64 was 59.8% in 2002, 58.9% in 2006, and 51.0% in 2010. Due to matters concerning consent for linking questionnaire data to registry information, only those from the 2002 cohort who participated in the first follow-up were included (46.1% of 2002 recruitment sample). Therefore, the total initial study sample was 69,401 participants, with 13,356 foreign-born immigrants (19.2%). Further exclusions were made based on criteria detailed in Fig. 1. Notable exclusions include all immigrants that arrived to Sweden under 18 years of age, as previous research has indicated that integration and health outcomes differ from those that immigrate as adults [43]. Additionally, all immigrants that have a potentially migration-related mental health diagnosis within 2 years of arriving to Sweden were excluded to help control for reverse causality (data retrieved from the National Board of Health and Welfare). Providing a description of the study sample, unweighted statistics of demographic and socioeconomic characteristics are presented in Table 1. Table 2 presents the weighted prevalences for social capital and psychological distress.

**Table 1:** Frequencies of the demographic and socioeconomic characteristics of the study sample

Descriptive characteristics	Swedish-born (*n* = 45,148)	Non-refugee (*n* = 2,643)	Refugee (*n* = 2,707)	Total (*n* = 50,498)
	%	%	%	%
Sex				
Men	45.8	41.1	52.0	45.9
Women	54.2	58.9	48.0	54.1
Age				
18–29	17.3	3.3	3.4	15.8
30–49	47.3	41.2	61.4	47.8
50–64	35.4	55.6	35.1	36.4
Education				
High	28.8	31.0	32.1	29.1
Middle	39.8	29.6	35.4	39.0
Low	31.4	39.4	32.5	31.9
Disposable family income				
Very high	23.3	17.3	10.4	22.3
High	23.0	21.2	15.2	22.5
Middle	20.5	20.4	17.5	20.3
Low	17.7	20.1	20.7	18.0
Very low	15.5	21.0	36.2	16.9
Family constellation				
Alone without children	17.2	16.8	15.3	17.1
Alone with children	1.3	1.9	2.4	1.4
Other adults without children	42.5	42.8	35.2	42.1
Other adults with children	39.0	38.5	47.1	39.4
Type of employment				
Permanent	65.6	63.3	56.4	65.0
Temporary	8.0	7.2	10.1	8.1
Self-employed	9.6	8.9	9.4	9.5
Retired, sick-leave, disability or activity pension	5.1	12.3	9.4	5.7
Leave of absense, student, trainee	6.5	2.8	5.3	6.3
Unemployed	2.4	2.7	6.4	2.6
Other	2.8	2.9	2.9	2.8
Occupational class				
Unskilled worker	14.9	22.7	32.1	16.3
Skilled worker	10.7	15.4	17.3	11.3
Low level salary	14.8	12.1	8.0	14.3
Medium level salary	27.2	23.5	19.0	26.5
High level salary	23.4	17.4	13.6	22.6
Self-employed	9.0	8.9	10.1	9.0

**Table 2:** Weighted prevalences of social capital and psychological distress of men and women, by immigrant status

Descriptive characterstics	Swedish-born	Non-refugee 3–9	Non-refugee 10–19	Non-refugee 20+	Refugee 3–9	Refugee 10–19	Refugee 20+	Total
M/W (n, sample)	20664/ 24484	202/ 259	205/ 306	679/ 992	264/ 327	622/ 568	522/ 404	23158/ 27340
M/W (n, weighted)	377067/ 348365	5220/ 5364	4979/ 6069	14114/ 17184	7354/ 7345	16680/ 12573	13653/ 9085	439067/ 405985
Bonding social capital	(%)	(%)	(%)	(%)	(%)	(%)	(%)	(%)
Social support								
High	90.1/ 93.7	79.9/ 86.3	78.7/ 86.8	77.1/ 87.4	64.1/ 72.4	69.4/ 75.2	68.4/ 77.7	87.5/ 91.9
Low	9.9/ 6.3	20.1/ 13.7	21.3/ 13.2	22.9/ 12.6	35.9/ 27.6	30.6/ 24.8	31.6/ 22.3	12.5/ 8.1
Bridging social capital								
Horizontal Trust								
High	91.2/ 91.4	79.4/ 85.0	84.8/ 86.1	83.6/ 88.7	69.8/ 71.4	71.0/ 75.0	74.5/ 81.1	89.1/ 90.0
Low	8.8/ 8.6	20.6/ 15.0	15.2/ 13.9	16.4/ 11.3	30.2/ 28.6	29.0/ 25.0	25.5/ 18.9	10.9/ 10.0
Horizontal Participation								
High	64.0/ 62.3	63.5/ 50.1	53.4/ 54.9	45.2/ 59.9	50.6/ 45.4	53.6/ 47.5	57.7/ 53.7	62.5/ 61.0
Low	36.0/ 37.7	36.5/ 49.9	46.6/ 45.1	54.8/ 40.1	49.3/ 54.6	46.4/ 52.5	42.3/ 46.3	37.5/ 39.0
Linking social capital								
Vertical trust								
High	70.0/ 74.5	59.3/ 65.0	52.5/ 63.3	58.0/ 64.5	58.0/ 57.7	54.7/ 52.6	58.7/ 54.7	68.2/ 72.3
Low	30.0/ 25.5	40.7/ 35.0	47.5/ 36.7	42.0/ 35.5	42.0/ 42.3	45.3/ 47.4	41.3/ 45.3	31.8/ 27.7
Vertical participation								
High	94.7/ 95.4	70.0/ 76.9	79.8/ 82.3	78.4/ 82.3	73.3/ 72.9	84.1/ 85.0	85.1/ 90.3	92.6/ 93.5
Low	5.3/ 4.6	30.0/ 23.1	20.2/ 17.7	21.6/ 17.7	26.7/ 27.1	15.9/ 15.0	14.9/ 9.7	7.4/ 6.5
Psychological distress								
No	83.1/ 74.7	76.0/ 75.9	77.2/ 74.2	81.2/ 81.1	72.3/ 70.7	75.0/ 67.7	83.1/ 74.7	82.3/ 74.6
Yes	16.9/ 25.3	24.0/ 24.1	22.8/ 25.8	18.8/ 18.9	27.7/ 29.3	25.0/ 32.3	22.9/ 29.1	17.7/ 25.4

### Outcome

#### Psychological distress

The General Health Questionnaire-12 (GHQ-12) [44], included in the SPHC questionnaires, was used to identify psychological distress and has demonstrated cross-cultural validity and reliability [45]. It was developed to capture symptoms that the participant experienced over the *past few weeks* with regards to, for example: sleep; the ability to concentrate and make decisions; and feelings of strain and worthlessness (see Appendix for full version). Answer options were of the variation: “more than usual”, “as usual”, “less than usual”, and “much less than usual”. Participants that answered 3 or more questions with either of the two dissenting options were designated as having “psychological distress” [44]. Participants that responded to at least 9 of the 12 questions were included, as there was little difference in the odds ratios for psychological distress between those who answered this subset versus those who answered the full set. The internal validity of GHQ-12 in this study sample was given by a Cronbach’s alpha of 0.88.

### Exposures

#### Immigrant status

“Immigrants” in this study were considered all individuals not born in Sweden. Immigrant status was based on the individual’s *reason for immigration* to Sweden. Two groups were formulated based on the participant’s country of birth and immigration year given by Statistics Sweden’s Total Population Register. Participants were designated as “refugees” whose country of birth and year of immigration match that of asylum-seekers to Sweden. “Non-refugees” were defined as those not from asylum-seeking countries. This proxy was used because the register information regarding a person’s reason for immigration is not reliable before 1998. Accordingly, all immigrants were categorised into these two groups, including individuals who came to Sweden for study or for family reunification. Throughout the rest of the paper, the term “immigrants” is used in reference to results that apply to all immigrants in the study (ie. to both refugees and non-refugees). The non-refugee and refugee groups were further stratified based on their *duration of residence* in Sweden, (3-9 years; 10-19 years; 20+ years). Individuals born in Sweden was the reference category and labelled “Swedish-born”. The register information for duration of residence in Sweden is based on the date that an individual’s residence permit is approved by the Migration Board and not from the immigrant’s date of entry into the country. Therefore some immigrants, particularly refugees, have been in Sweden longer than is noted in our dataset.

### Potential mediators

*Social capital* was measured using five questions on bonding, bridging and linking social capital from the SPHC questionnaire. These questions are variations of social capital indicators that have been theoretically validated and widely used with consistent results [24, 25, 46–48]. Both cognitive and structural components were measured for bridging and linking social capital, whereas only the cognitive component was measured for bonding social capital. All social capital variables were dichotomized.

*Bonding social capital* was determined by answers to the question: “Do you know any people who can provide you with personal support for personal problems or crises in your life?” Possible answers were: “Yes, always”, “yes, for the most part”, “no, usually not”, “no, never”. The first two options were designated as “high social support” whereas the last two were designated as “low social support”.

*Bridging social capital* refers to two variables— horizontal trust and horizontal participation. *Horizontal trust* was measured by the statement: “You can trust most people living in this neighbourhood.” The four response alternatives were “very accurate”, “fairly accurate”, “not particularly accurate” and “not at all”. The first two options were designated as “high horizontal trust”, while the last two were designated as “low horizontal trust”. *Horizontal participation* was measured by the question: “In the past 12 months, have you more or less regularly participated in activities together with several other people? (For example sport, music/theatre, courses, religious gatherings, choir, sewing groups, political associations or other society).” Possible answers were “yes” or “no” and designated as “high horizontal participation” and “low horizontal participation”.

*Linking social capital* refers to two variables— vertical trust and vertical participation. *Vertical trust* was measured by the following question: “How much confidence do you have in the following public institutions?: medical services; the police; the parliament; politicians in your municipality.” Each item falls into one of two aspects of political trust – trust in the executive branch (first two items) and trust in the legislative branch (last two items). Initially, to be included in the analysis responders had to answer all four items. This result did not differ significantly from those who answered only one of each aspect, which was therefore used to preserve power. The response alternatives for each item were “considerable”, “fairly considerable”, “little”, “none whatsoever”—which were given a value from 4 to 1 —and “have no opinion” which was treated as a missing value. The variable was dichotomized by adding the scores of each item and dividing it by the number answered. Those that fell at or above 2.5 were designated as “high vertical trust” and those that fell below 2.5 designated “low vertical trust”. *Vertical participation* was measured by the question: “Did you vote in any of the political elections 2002?” “Yes” was designated as “high vertical participation”, and “no”, as “low vertical participation”.

### Confounders

#### Demographic and socioeconomic variables

*Age* was analysed as a continuous variable. *Educational level was* trichotomised— high: university degree; medium: completion of secondary education; low: only compulsory education, vocational training, or some secondary education. *Disposable family income* was divided into quintiles representing the annual disposable income of a household after taking into account expenses related to taxes, family size and constellation. *Occupational position* (high level salaried employees; intermediate level salaried employees; low level salaried employees; skilled workers; unskilled workers; self-employed) is a measure of class based on occupation [49]. *Type of Employment* was assessed by the question “Which of the following alternatives apply to you right now?” Answers were divided into seven categories: permanent employment; temporary employment; own business or business partner; retired, on sick leave, disability, or receiving activity benefit; on leave of absence or student or trainee; unemployed; and other. *Family constellation* was divided into four categories: living alone with children; living alone without children; living with other adult and children; living with other adult without children. The first three variables, along with *sex*, were taken from the Longitudinal Integration Database for Health Insurance and Labour Market Studies provided by Statistics Sweden, while the last two were taken from the questionnaire.

### Statistical analysis

Baron and Kenny [50] outline four steps to determine the mediating role of a variable. This procedure was used to investigate if social capital mediates the association between immigrant status and psychological distress. The steps are: first, if immigrant status is associated with psychological distress; second, if immigrant status is associated with social capital; third, if social capital is associated with psychological distress; and fourth, if adjusting for social capital partially or completely attenuates the association of immigrant status and psychological distress. These conditions were tested using weighted logistic regression, stratified by sex and duration of residence in Sweden. Each social capital indicator was tested separately for steps two and three to establish their associations and were adjusted for demographic and socioeconomic factors. As our main interest is the mediatory role of bonding, bridging and linking social capital, the primary focus of this study is on steps one and four.

The tests for the first and fourth steps are presented with a number of models. For step one, the age-adjusted estimates of immigrant status on psychological distress are shown in Model 1. Estimates are adjusted for demographic and socioeconomic variables in Model 2 and those immigrant groups with significant associations to psychological distress after this adjustment, are considered eligible for mediation by social capital. For step four, bonding, bridging (horizontal trust and participation), and linking social capital (vertical trust and participation) were added to Model 2 separately (Model 3a, 3b, 3c) and together (Model 4). All results are presented as Odds Ratios (OR) with 95% confidence intervals (CI), using SAS 9.3.

Additionally, for immigrant groups that were eligible for social capital mediation, the Sobel test was used to assess if the mediated effect of each individual social capital variable was significant [50, 51]. Furthermore, the Sobel test was used to test the multiple mediation effect for two (Models 3b-c) and five (Model 4) social capital variables.

## Results

A total of 50,498 individuals were included in this study, 10.6% of whom were immigrants. Table 1 shows descriptive statistics for the demographic and socioeconomic characteristics. Refugees constituted 50.6% of immigrants. Overall, women were more represented than men, except in the refugee category, for which men comprised 52%. Table 2 shows prevalences for social capital and psychological distress. Approximately 18% of men and 25% of women reported having psychological distress. There was little difference in the prevalence of psychological distress between non-refugee men and women. Refugee men in Sweden 10–19 and 20+ years reported psychological distress less than women of the same category. Prevalences for non-refugee women were lower or nearly equivalent to Swedish-born women, whereas refugee women had higher prevalences. Both non-refugee and refugee men had higher prevalences of psychological distress compared to Swedish-born men. Overall, refugees reported more psychological distress compared to non-refugees. The occurrence of psychological distress peaked at 10-19 years in Sweden for immigrant women, whereas it progressively decreased for immigrant men.

All immigrant categories reported worse social capital compared to Swedish-born and was generally lower for men compared to women, with a few exceptions. Non-refugee women in Sweden 3-9 years and all categories of refugee women reported worse horizontal participation than men in the same category. Additionally, refugee women reported slightly worse vertical trust. Social capital was generally progressively better for immigrants with longer residence. However, vertical trust was lowest for immigrants in Sweden 10-19 years and horizontal participation was progressively worse among non-refugee men with longer duration of residence.

Table 3 presents results for the association between immigrant status and psychological distress for men. The first step of mediation is shown in Models 1 and 2. The age-adjusted ORs (Model 1) showed significantly higher odds of psychological distress for all immigrant men compared to their Swedish-born counterparts. Adjusting for socioeconomic variables (Model 2) decreased point estimates slightly for non-refugees in the 3-9 years category, demonstrating no significant difference compared to Swedish-born. Point estimates increased slightly for non-refugees in Sweden 10-19 years but decreased 32-39% for non-refugees in Sweden 20+ years and all refugee categories, though estimates were still significant. The odds of psychological distress were greater for refugees than for non-refugees. All immigrant groups except non-refugees in Sweden 3-9 years were eligible for mediation.

**Table 3:** Odds ratios with 95% confidence intervals of the association between immigrant status and psychological distress for immigrant men in 4 models

Immigrant status	Model 1	Model 2	Model 3a	Model 3b	Model 3c	Model 4
Swedish-born	1	1	1	1	1	1
Non-refugee 3–9	**1.43 (1.00–2.04)**	1.34 (0.92–1.93)	1.19 (0.81–1.73)	1.23 (0.84–1.78)	1.28 (0.88–1.86)	1.11 (0.75–1.62)
Non-refugee 10–19	**1.55 (1.08–2.23)**	**1.58 (1.09–2.29)**	1.40 (0.96–2.03)	**1.47 (1.01–2.13)**	**1.46 (1.01–2.13)**	1.28 (0.88–1.86)
Non-refugee 20+	**1.70 (1.37–2.13)**	**1.45 (1.15–1.83)**	**1.31 (1.04–1.66)**	**1.35 (1.07–1.71)**	**1.41 (1.11–1.78)**	1.24 (0.98–1.58)
Refugee 3–9	**1.83 (1.35–2.48)**	**1.56 (1.13–2.15)**	1.20 (0.87–1.67)	1.35 (0.99–1.85)	**1.51 (1.10–2.08)**	1.10 (0.79–1.52)
Refugee 10–19	**1.83 (1.50–2.24)**	**1.63 (1.33–2.00)**	**1.35 (1.09–1.66)**	**1.41 (1.15–1.74)**	**1.55 (1.26–1.91)**	1.20 (0.96–1.49)
Refugee 20+	**2.01 (1.60–2.52)**	**1.62 (1.28–2.04)**	**1.33 (1.06–1.68)**	**1.44 (1.14–1.82)**	**1.55 (1.24–1.95)**	1.21 (0.96–1.53)

Model 1 adjusted for age Model 2 is as model 1 plus adjustment for socioeconomic factors (occupational class, disposable family income, education, type of employment, and family constellation) Model 3a and 3b and 3c as model 2 with additional adjustment for bonding social capital, bridging social capital (horizontal trust and horizontal participation), and linking social capital (vertical trust and vertical participation), respectively Model 4 is as model 2 with additional adjustment for all social capital variables in Models 3a-c Significant associations are in bold

For women, age-adjusted estimates (Table 4, Model 1) showed no significant difference in psychological distress between Swedish-born women, non-refugees (regardless of duration of residence), and refugees in Sweden 3-9 years. Refugees who resided in Sweden for 10-19 years and 20+ years respectively had 54% and 75% higher odds of psychological distress than Swedish-born women. After adjusting for socioeconomic variables (Model 2), point estimates decreased slightly for both non-refugees and refugees in Sweden 3-9 years and non-refugees in Sweden 20+ years. These adjustments slightly increased estimates for non-refugees and refugees in Sweden 10-19 years and had the greatest effect on ORs for refugees in Sweden 20+ years, decreasing point estimates by 23%. Refugee status remained significantly associated with psychological distress for those who had resided in Sweden 10 years or longer and were therefore eligible for mediation.

**Table 4:** Odds ratios with 95% confidence intervals of the association between immigrant status and psychological distress for immigrant women in 4 models

Immigrant status	Model 1	Model 2	Model 3a	Model 3b	Model 3c	Model 4
Swedish-born	1	1	1	1	1	1
Non-refugee 3–9	0.85 (0.62–1.16)	0.84 (0.61–1.15)	0.76 (0.54–1.05)	0.79 (0.57–1.09)	0.76 (0.55–1.05)	**0.68 (0.49–0.95)**
Non-refugee 10–19	1.09 (0.82–1.46)	1.16 (0.86–1.56)	1.08 (0.79–1.46)	1.11 (0.82–1.49)	1.07 (0.79–1.45)	0.98 (0.72–1.34)
Non-refugee 20+	1.05 (0.88–1.26)	1.00 (0.83–1.20)	0.95 (0.79–1.15)	0.98 (0.81–1.18)	0.94 (0.78–1.13)	0.89 (0.74–1.08)
Refugee 3–9	1.12 (0.86–1.46)	1.10 (0.84–1.45)	0.86 (0.65–1.14)	0.97 (0.73–1.27)	0.99 (0.75–1.30)	**0.73 (0.54–0.98)**
Refugee 10–19	**1.54 (1.26–1.87)**	**1.57 (1.28–1.92)**	**1.29 (1.05–1.60)**	**1.41 (1.15–1.73)**	**1.41 (1.15–1.73)**	1.12 (0.91–1.39)
Refugee 20+	**1.75 (1.36–2.24)**	**1.58 (1.22–2.04)**	**1.36 (1.05–1.77)**	**1.48 (1.15–1.91)**	**1.47 (1.14–1.88)**	1.24 (0.96–1.61)

Model 1 adjusted for ageModel 2 is as model 1 plus adjustment for socioeconomic factors (occupational class, disposable family income, education, type of employment, and family constellation)Model 3a and 3b and 3c as model 2 with additional adjustment for bonding social capital, bridging social capital (horizontal trust and horizontal participation), and linking social capital (vertical trust and vertical participation), respectively Model 4 is as model 2 with additional adjustment for all social capital variables in Models 3a-cSignificant associations are in bold

Table 5 shows the results for the second step of the mediation procedure— if immigrant status is associated with social capital. After adjustment for socioeconomic factors, immigrant status was significantly associated with all social capital indicators, except for nearly half of the estimates for horizontal participation. The results demonstrated a tendency toward decreased odds of low social capital the longer an individual was in Sweden, though this was not universally true.

**Table 5:** Odds ratios with 95% confidence intervals of the association between immigrant status and low social capital. All results are presented separately for men and women and adjusted for demographic and socioeconomic factors (age, occupational class, disposable family income, education, type of employment, and family constellation)

Social capital	Bonding social capital	Bridging social capital		Linking social capital	
Variables	Social support	Horizontal trust	Horizontal participation	Vertical trust	Vertical participation
Immigrant status					
Men					
Swedish-born	**1**	**1**	**1**	**1**	**1**
Labour 3–9	**2.57 (1.74–3.78)**	**2.96 (1.93–4.54)**	1.17 (0.84–1.64)	**1.79 (1.29–2.48)**	**9.94 (6.81–14.5)**
Labour 10–19	**2.59 (1.77–3.79)**	**2.39 (1.56–3.66)**	**1.45 (1.06–2.00)**	**2.24 (1.62–3.11)**	**5.98 (3.92–9.13)**
Labour 20+	**1.92 (1.55–2.37)**	**2.26 (1.74–2.93)**	**1.28 (1.08–1.53)**	**1.43 (1.19–1.71)**	**4.70 (3.70–5.98)**
Refugee 3–9	**4.68 (3.45–6.34)**	**3.69 (2.68–5.08)**	**1.55 (1.18–2.04)**	**1.50 (1.12–1.99)**	**5.53 (3.95–7.73)**
Refugee 10–19	**3.48 (2.83–4.28)**	**4.23 (3.4–5.27)**	**1.25 (1.04–1.49)**	**1.74 (1.45–2.10)**	**3.08 (2.37–4.01)**
Refugee 20+	**3.19 (2.55–4.00)**	**4.29 (3.38–5.43)**	0.87 (0.71–1.07)	**1.55 (1.26–1.91)**	**3.26 (2.41–4.42)**
Women					
Swedish-born	**1**	**1**	**1**	**1**	**1**
Labour 3–9	**2.57 (1.70–3.87)**	**1.78 (1.21–2.62)**	**1.66 (1.26–2.18)**	**1.67 (1.24–2.26)**	**6.92 (4.81–9.94)**
Labour 10–19	**2.18 (1.47–3.23)**	**2.08 (1.40–3.08)**	1.26 (0.97–1.63)	**1.80 (1.37–2.38)**	**5.71 (3.90–8.36)**
Labour 20+	**1.53 (1.22–1.93)**	**1.71 (1.35–2.17)**	0.97 (0.84–1.13)	**1.43 (1.23–1.67)**	**6.24 (4.98–7.81)**
Refugee 3–9	**5.50 (4.12–7.36)**	**3.86 (2.94–5.08)**	**1.69 (1.32–2.17)**	**2.08 (1.62–2.69)**	**7.12 (5.21–9.72)**
Refugee 10–19	**4.20 (3.32–5.32)**	**3.74 (2.94–4.76)**	**1.49 (1.22–1.81)**	**2.53 (2.08–3.09)**	**3.73 (2.79–5.00)**
Refugee 20+	**2.95 (2.20–3.97)**	**2.79 (2.06–3.78)**	1.15 (0.91–1.45)	**2.06 (1.63–2.61)**	**2.55 (1.71–3.80)**

Significant associations are in bold

Regarding step three of mediation [results not shown, see “Additional file 1”], social capital variables generally showed a significant association with psychological distress for immigrant categories where mediation was considered possible (ie. where associations in Model 2 of Tables 3 and 4 were significant). Exceptions included: vertical participation for both men and women; horizontal participation for immigrant men except non-refugees in Sweden 10-19 years, for which the association was significant; vertical trust for non-refugee men in Sweden 20+ years; and horizontal trust for refugee women in Sweden 20+ years.

The fourth step of the mediation procedure — testing the attenuation of the association between immigrant status and psychological distress by social capital— is shown in Tables 3 and 4. For men (Table 3), at least some of the effect demonstrated in Model 2 was decreased after adjusting for each type of social capital (Models 3a-c). For non-refugees in Sweden 10-19 years and refugees in Sweden 3-9 years, bonding social capital fully explained the association with psychological distress, as did bridging social capital for refugees in Sweden 3-9 years. Bonding social capital (Model 3a) had the largest mediatory effect on the association between immigrant status and psychological distress compared to bridging or linking social capital. After adjusting for all social capital variables together (Model 4), the ORs for all eligible immigrant groups became insignificant. The Sobel tests (results not shown) for one, two and five variable mediation were significant for all eligible immigrant groups, except for bonding and bridging social capital for non-refugee men in Sweden 20+ years and refugee men in Sweden 3–9 years.

Regarding women (Table 4), adjusting for each social capital variable (Models 3a-c) also decreased the effect shown in Model 2. Bonding social capital (Model 3a) decreased the associations the most, though refugees in the 10-19 and 20+ years categories still showed significantly increased odds of psychological distress compared to Swedish-born. Adjusting for all the mediatory variables together (Model 4) demonstrated the greatest reduction in ORs, bringing estimates of immigrants in Sweden 10-19 and 20+ years to insignificance. Additionally, psychological distress for immigrants in Sweden 3-9 years became significantly lower compared to Swedish-born. The Sobel tests (results not shown) demonstrated significant mediation effects for bonding, bridging and linking social capital and the five variable combination for all eligible immigrant groups.

## Discussion

This study investigated mental health inequalities between foreign-born immigrants and Swedish-born and whether social capital explained these differences. The results demonstrated that psychological distress does vary based on reason for immigration, duration of residence in Sweden and gender. Our main findings show that refugee women who have resided in Sweden at least 10 years and all immigrant men have worse mental health than Swedish-born. Furthermore, social capital explained differences between these immigrant groups and Swedish-born. Bonding social capital was the most important among the three social capital variables, although together they demonstrated the strongest effect. The age-adjusted estimates showed that refugees had greater risk of psychological distress than non-refugees when compared to their Swedish-born counterparts. After adjusting for socioeconomic factors, immigrant men had significantly worse mental health than Swedish-born, except for non-refugees in Sweden less than 10 years. Conversely, there was no significant difference between non-refugee women and their Swedish-born counterparts. Only refugee women in Sweden for at least 10 years had worse mental health than their Swedish-born counterparts. These results partially support our first hypothesis. In accordance with the findings of a 2013 Swedish register-based study [36], our prevalence estimates demonstrate that women do have worse mental health compared to men, while the regression analysis shows that when compared to their Swedish-born counterparts, men are worse off.

A number of factors may make immigrant men, and refugee women in Sweden 10 or more years, particularly vulnerable to psychological distress. First, refugee men and even non-refugee men are often the first to come to Sweden to apply for asylum or work, respectively. Requests to be reunited with their families are granted sometime later. Thus some immigrant men may lack social support systems important for maintaining mental health in a new country. Second, refugee men are more likely to experience adverse pre-migration events that might affect mental health, such as internment in refugee camps, torture, imprisonment or combat [8, 52]. However, at least some of this effect was removed by excluding immigrants who had a migration-related mental health diagnosis within two years of arriving to Sweden. Third, previous research has demonstrated a discrepancy between individual's expectations for themselves related to their gender and the realization of those expectations. For example, a study of Ethiopian and Eritrean refugees in North America found that men perceived the resettlement process as being accompanied by a decrease in status due to changing gender roles, whereas women perceived this as a means of greater opportunity [53]. The same study found that men perceived fewer occupational opportunities compared to in their native countries, while women in the same group perceived the opposite. This, coupled with the fact that women are more likely to come to Sweden with some means of established social support, may contribute to the lack of psychological distress seemingly experienced by refugee women in the first 9 years after arrival. Leading to the fourth point, finding a job immediately may be more crucial for men than for women. A Norwegian study found that although lack of paid employment was associated with psychological distress for both immigrant men and women, it was more profound for men [52]. The 2008 Swedish government's strategy for integration reports that the median time for refugees to find employment is 7–8 years after being granted residency [54]. Although it takes refugee men fewer years to find employment in Sweden than refugee women [54], the results of this study show that newly-arrived refugee men (3-9 years) have significantly higher psychological distress than their Swedish-born counterparts while newly-arrived refugee women do not. Long periods of unemployment together with vulnerability due to a potentially greater expectation-reality discrepancy, could also explain why adjusting for socioeconomic factors, which included employment status, accounts for a third of the odds of psychological distress for all refugee men and most non-refugee men, whereas it makes little difference for refugee women. Furthermore, refugee men showed no differences in risk of psychological distress according to years in Sweden. Conversely, point estimates for refugee women and non-refugee men and women indicated that immigrants who lived in Sweden 10-19 years are particularly vulnerable to psychological distress, contrary to our second hypothesis. This could reflect a cohort effect, as obtaining employment for immigrants who lived in Sweden 10-19 years was particularly challenging given that, for many, their arrival coincided with Sweden’s recession in the 1990s [55]. The considerable jump between the risk of psychological distress for refugee women in Sweden 3-9 years compared to 10-19 years could be influenced by the fact that the median time it takes for refugee women to find employment in Sweden is 10 years [54].

Where controlling for socioeconomic variables only accounted for part of the differences in psychological distress, adjusting for social capital indicators accounted for the rest, supporting our third hypothesis. Since the majority of refugees in our sample are from non-western origins, this supports the finding of Tinghög and colleagues [56] that socioeconomic differences only partially explain the increased risk for depression of non-Western immigrants compared to Swedish-born. The final point estimates demonstrated some over-risk for all immigrant men and for some groups of refugee women, though none were significant.

Previous studies give little support for social capital explaining the relationship between socioeconomic status and mental health inequalities specifically [14, 16, 17, 27, 57]. However, there is some evidence that social capital may explain differences between immigrant or ethnic groups [14, 16, 17, 58]. Of the three social capital indicators in the present study, bonding social capital had the greatest mediatory effect. This can be explained by social support characterising strong ties, which is expected to have a more profound impact on mental health than the weaker ties that characterise bridging or linking social capital. While these findings support previous research [14, 17, 58], it contrasts with the results of a Finnish study that determined that the association of trust and participation with psychological health explained nearly all the effect of social support [28].

Though investigating differences in the mediatory effect of social capital according to gender was beyond the scope of this paper, previous studies have indicated that there could be variations in the importance of different forms of social capital for the health of men and women [30, 59]. In addition to accounting for differences between groups, the mediation also uncovered differences between Swedish-born and immigrant women (both non-refugee and refugee) that were most recently arrived (3-9 years). This further demonstrates that the expectations and conditions of newly-arrived immigrant women may contribute to a greater perception of mental well-being than Swedish-born women.

Given that social capital demonstrates a mediatory effect, the fact that it increases with duration of residence while mental health outcomes are generally worse for immigrants in Sweden 10 years or more presents a paradox. Though this study’s design does not allow for directly investigating the relationship between social capital and mental health over-time, there may be two possible explanations. One may be a cohort effect reflecting the differences in the economic and social environment between years or variations in how individuals in a cohort might perceive and report mental health. Another may be that duration of residence itself is a modifier of the effect of social capital on mental health, as previous studies have indicated that the earlier immigrants acquire social capital, the better their mental health [35, 60, 61]. For example, a Swedish study found that the social capital of newly-arrived Iraqi refugees protected against the detrimental effects of integration-related stressors on mental health [60]. A Canadian study found that the combined effect of being a newly-arrived immigrant and having low social support greatly increased the risk of reporting anxiety or mood disorders [61]. To investigate this latter possibility, an additive interaction analysis was conducted to see if social capital has a greater effect on mental health the longer an immigrant has been in Sweden. For refugees, only the interaction between bonding social capital and refugees in Sweden 20+ years was significant, though the results demonstrated a general trend towards increased effect modification for all social capital variables with increased duration of residence. While there were no significant interaction effects for non-refugees, bonding and linking social capital was greater for those in Sweden 10-19 years than for those in Sweden 20+. However, these results are difficult to interpret due to wide confidence intervals. Further research is needed to investigate the effect of social capital on mental health years later.

### Strengths and limitations

One strength of this study is that it does not follow the conventional classification of immigrants based solely on region of origin. There is great heterogeneity in immigrant factors that might affect mental health. A register-based Swedish study by Hollander and colleagues [36] showed that Asian, Iraqi, and Middle Eastern refugees had worse mental health than non-refugees from the same origins. Therefore, the distinction between refugees and non-refugees is important. As register information on an individual’s reason for immigration is not reliable before 1998, refugees in this study were categorized as “non-refugee” and “refugee” based on their country of birth and year of immigration. Consequently, this included individuals with other migration claims, of which those who seek family reunification are a particularly large group [62]. It can be argued that it is reasonable to classify them as non-refugee or refugee based on the assumption that their pre-migration exposures and post-migration opportunities resemble that of others immigrating from the same time and place. However, as discussed above, they do often arrive into circumstances with better social support, and may underestimate our results. Additionally, some individuals that came to Sweden on study permits stayed permanently and could be included in this sample. Classifying students as non-refugees is not expected to affect these results whereas designating them as refugees, may underestimate the results. However, as statistics from the Swedish Migration Board indicate that students made up only 5% of the total immigrant population between 1980 and 2007 [63], it is not expected to greatly affect the results. Misclassification also occurred for some refugees regarding their duration of residence in Sweden, as date of residence is based on date of registration, not date of entry to Sweden. This would reduce the differences in psychological distress between duration of residence groups, but is not believed to significantly alter the results.

Most migration health studies focus on one specific type of immigrant group (eg. refugees), while the large sample size of this study allowed for comparing groups. Additionally, these groups were further stratified by years in Sweden, a factor not often taken into account due to power constraints and multicollinearity with indicators of immigrant origin. Instruments for assessing social capital and mental health in this study have been widely validated, though possibly not for all cultural contexts represented in this sample. All mental health and social capital measures were self-reported at the same point in time. Therefore misclassification may have occurred due to mood-congruent attentional bias [64], resulting in respondents with psychological distress portraying that they also have low social capital. Some previous research has also suggested that social capital may be particularly low in totalitarian societies, where trusting institutions, fellow citizens or participating in civic life may not be rational. However, social support from friends and relatives is likely strong and important even in these contexts. Many refugees in this study come from such countries and this could have repercussions for the relative importance of different forms of social capital for the health and wellbeing of people of different origins [65]. Therefore, when studying the contribution of different forms of social capital for health inequalities by immigrant status, it has to be taken into consideration that the associations may also reflect patterns of social capital and health in the country of origin and not merely exposures in the country of destination (i.e. Sweden). This is particularly relevant with regards to our measure of bonding social capital, as it does not distinguish whether one has individuals that lend support who live in Sweden or elsewhere, whereas bridging and linking social capital questions are more clearly interpreted as bound in Sweden.

Reverse causality is an inherent challenge with cross-sectional studies. In this study, causal directionality is preserved between the exposure and the mediator and the exposure and the outcome, as immigration occurred before the measurement of social capital and mental health. However, reverse causality is potentially reflected in the association between social capital and psychological distress. To eliminate at least some of the potential effect of mental health on social capital, we excluded immigrants who were diagnosed with a potentially migration-related mental illness within two years of arrival to Sweden. Furthermore, though refugees in particular may experience pre-migration circumstances that might negatively impact their post-migration mental health, studies are not consistent as to the significance of the enduring effects it may have [9, 40, 66]. Additionally, our own results demonstrate that mental health seems to be better for those who are newly arrived. This is in line with previous research supporting the “healthy immigrant effect” in which newly arrived immigrants are healthier than the host population, but for which health decreases over time [38]. Based on these considerations, we believe that reverse causality is not largely reflected in our results.

Another limitation of this study is the selection bias introduced through a lower response rate among individuals with lower social capital and among those with psychological distress. Furthermore, given that only 10.6% of our study sample is immigrants and the foreign-born population of Stockholm, excluding by similar eligibility criteria, was 18–19% in 2002, 2006 and 2010 [personal communication between KE and Statistics Sweden: Email, subject: Utländsk bakgrund, 2 February 2015], immigrants are underrepresented in our study, with some groups potentially not represented at all. To account for immigrant underrepresentation, the 2006 cohort questionnaires were translated into six languages [41] and our analysis uses statistical methods of reweighting for non-response based on country of birth [41, 67]. The remaining selection bias would affect prevalence estimates, while the odds ratios regarding immigration, social capital and psychological distress are not expected to be largely affected [68].

## Conclusion

Immigrant status may be one aspect of an immigrant’s social position in a new society that can impact their mental health. To our knowledge, this is the first study to look at immigrant status by using reason for immigration and duration of residence together as main exposures for mental health. The results of this study demonstrate that social capital explains mental health inequalities for some immigrant groups in relation to the Swedish-born population. Furthermore, these findings also indicate that foreign-born immigrants with persistently low social capital over time may be particularly vulnerable to psychological distress. This highlights social capital as a potentially important post-migration factor for promoting mental health, and therefore is a consideration for policy-makers and researchers. The cross-sectional nature of this study limits the possibility to draw strong conclusions on causality from our findings. Longitudinal studies are needed to further investigate these observations.

## Appendix

### General Health Questionnaire-12 as formulated in the Stockholm Public Health Cohort

Have you been able to concentrate on what you are doing in the *past few weeks*?

- Better than usual
- As usual
- Worse than usual
- Much worse than usual

Have you lost much sleep over worry in the *past few weeks*?

- Not at all
- Not more than usual
- More than usual
- Much more than usual

Have you felt that you are playing a useful part in things in the *past few weeks*?

- More than usual
- As usual
- Less than usual
- Much less than usual

Have you felt capable of making decision about things in the *past few weeks*?

- More than usual
- As usual
- Less than usual
- Much less than usual

Have you felt constantly under strain in the *past few weeks*?

- Not at all
- Not more than usual
- More than usual
- Much more than usual

Have you felt that you could not overcome your difficulties in the *past few weeks*?

- Not at all
- Not more than usual
- More than usual
- Much more than usual
- Much more than usual

In the *past few weeks*, have you been able to enjoy your normal day to day activities?

- More than usual
- As usual
- Less than usual
- Much less than usual

Have you been able to face up to your problems in the *past few weeks*?

- More than usual
- As usual
- Less than usual
- Much less than usual

Have you been feeling unhappy or depressed in the *past few weeks*?

- Not at all
- Not more than usual
- More than usual
- Much more than usual

In the *past few weeks*, have you been losing confidence in yourself?

- Not at all
- Not more than usual
- More than usual
- Much more than usual

Have you been thinking of yourself as a worthless person in the *past few weeks*?

- Not at all
- Not more than usual
- More than usual
- Much more than usual

Have you been reasonably happy in the *past few weeks*, all things considered?

- More than usual
- As usual
- Less than usual
- Much less than usual

## Additional file

Additional file 1: Odds ratios with 95% confidence intervals of the association between social capital and psychological distress, stratified by immigrant status. The results for men and women are presented separately. (PDF 322 kb)
