# Supplementary material for: The role of social capital in explaining mental health inequalities between immigrants and Swedish-born: a population-based cross-sectional study
Source: BMC Public Health. 2017 Jan 25;17:117. doi: 10.1186/s12889-016-3955-3 (PMC5264487; doi:10.1186/s12889-016-3955-3)
Supplement: Additional file 1: — Odds ratios with 95% confidence intervals of the association between social capital and psychological distress, stratified by immigrant status. The results for men and women are presented separately. (PDF 322 kb) [file 12889_2016_3955_MOESM1_ESM.pdf]

Additional file 1: Baron and Kenny mediation analysis, step 3 results

Johnson et al., 2017

Supplementary Table 1: Odds ratios with 95% confidence interval (CI) of the association between social capital and poor mental health, for **men**. Results are stratified by immigrant status. Bolded estimates indicate where the association is not significant.

Social Capital	Swedish-born	Non-refugee 3-9	Non-refugee 10-19*	Non-refugee 20+*	Refugee 3-9*	Refugee 10-19*	Refugee 20+*
**Bonding social capital**							
Social support							
High	1	1	1	1	1	1	1
Low	2.74 (2.43-3.09)	**1.80 (0.72-4.54)**	2.95 (1.22-7.17)	3.67 (2.19-6.16)	2.60 (1.39-4.87)	1.84 (1.19-2.84)	3.52 (2.10-5.89)
**Bridging social capital**							
Horizontal Trust							
High	1	1	1	1	1	1	1
Low	1.75 (1.52-2.01)	N/R**	2.93 (1.05-8.21)	2.31 (1.30-4.11)	5.15 (2.49-10.65)	2.01 (1.29-3.11)	2.77 (1.59-4.81)
Horizontal Participation							
High	1	1	1	1	1	1	1
Low	1.28 (1.17-1.40)	**1.64 (0.71-3.82)**	**1.58 (0.69-3.60)**	1.91 (1.16-3.15)	**1.31 (0.68-2.53)**	**1.32 (0.86-2.04)**	**1.52 (0.93-2.47)**
**Linking social capital**							
Vertical trust							
High	1	1	1	1	1	1	1
Low	1.54 (1.41-1.69)	**1.19 (0.54-2.63)**	2.45 (1.09-5.50)	**1.48 (0.93-2.37)**	3.05 (1.54-6.06)	2.49 (1.58-3.93)	3.29 (1.94-5.58)
Vertical participation							
High	1	1	1	1	1	1	1
Low	**1.03 (0.85-1.25)**	**0.70 (0.31-1.58)**	**1.41 (0.54-3.66)**	**0.92 (0.53-1.60)**	**1.26 (0.63-2.52)**	**1.01 (0.58-1.77)**	**0.60 (0.28-1.28)**

*Immigrant categories eligible for mediation
**No results due to small stratum sample size
All ORs adjusted for demographic and socioeconomic factors (age, occupational class, income, education, type of employment, and family constellation).

Supplementary Table 2: Odds ratios with 95% confidence interval (CI) of the association between social capital and poor mental health, for **women**. Results are stratified by immigrant status. Bolded estimates indicate where the association is not significant.

Social Capital	Swedish-born	Non-refugee 3-9	Non-refugee 10-19	Non-refugee 20+	Refugee 3-9	Refugee 10-19*	Refugee 20+*
**Bonding social capital**							
Social support							
High	1	1	1	1	1	1	1
Low	3.18 (2.80-3.61)	3.23 (1.29-8.12)	2.54 (1.03-6.31)	4.22 (2.66-6.68)	2.77 (1.51-5.06)	1.94 (1.21-3.10)	3.3 (1.85-5.90)
**Bridging social capital**							
Horizontal Trust							
High	1	1	1	1	1	1	1
Low	1.72 (1.53-1.93)	**1.74 (0.64-4.73)**	**1.77 (0.73-4.30)**	2.28 (1.40-3.70)	**1.23 (0.68-2.21)**	1.58 (1.00-2.50)	**1.73 (0.89-3.35)**
Horizontal Participation							
High	1	1	1	1	1	1	1
Low	1.29 (1.20-1.38)	**1.25 (0.59-2.62)**	**1.66 (0.87-3.16)**	**1.33 (0.91-1.95)**	**1.36 (0.77-2.41)**	1.79 (1.17-2.75)	2.22 (1.31-3.79)
**Linking social capital**							
Vertical trust							
High	1	1	1	1	1	1	1
Low	1.58 (1.46-1.70)	3.32 (1.60-6.93)	**1.84 (0.89-3.83)**	1.95 (1.37-2.76)	**1.09 (0.63-1.88)**	1.60 (1.03-2.49)	2.48 (1.44-4.26)
Vertical participation							
High	1	1	1	1	1	1	1
Low	1.25 (1.07-1.46)	**1.00 (0.44-2.25)**	**0.88 (0.40-1.94)**	**1.22 (0.78-1.92)**	**1.73 (0.91-3.28)**	**0.92 (0.50-1.69)**	**2.29 (0.93-5.60)**

*Immigrant categories eligible for mediation.
All ORs adjusted for demographic and socioeconomic factors (age, occupational class, income, education, type of employment, and family constellation).
